# Structure–Function Engineering of Lignin-Based Hydrogels for Adsorptive Removal of Organic Dyes and Heavy Metal Ions: A Category-Oriented Review

**DOI:** 10.3390/gels12080688

**Published:** 2026-08-03

**Authors:** Jianhui Guo, Yue Hu, Yiming Sun, Chang Ma, Minghui Zhang, Yida Niu, Youming Dong, Cheng Li

**Affiliations:** 1 Department of Environmental Design, College of Fine Arts, College of Foreign Languages, Henan University, Kaifeng 475001, China; 13663022485@163.com (Y.H.); 104753232022@henu.edu.cn (Y.S.); machang2016@163.com (C.M.); 18790325382@163.com (M.Z.); 2 College of Art, Zhengzhou University of Science and Technology, Zhengzhou 450064, China; niuyida@zit.edu.cn; 3 College of Materials Science and Technology, Nanjing Forestry University, Nanjing 210037, China; youming.dong@njfu.edu.cn; 4 College of Forestry, Henan Agricultural University, Zhengzhou 450046, China

**Keywords:** lignin, hydrogel adsorbents, heavy metal ions, organic dyes, adsorptive removal, wastewater treatment

## Abstract

Given the widespread contamination of water bodies by diverse pollutants, particularly heavy metal ions and organic dyes, there is an urgent need to develop efficient and sustainable biomass adsorbents. Lignin is rich in active groups such as phenolic hydroxyl and carboxyl groups, making it a natural adsorbent. However, its application is still hindered by limitations, including restricted solubility and low reactivity. Converting lignin into three-dimensional porous hydrogels not only overcomes the inherent structural brittleness of lignin-based materials but also accelerates the diffusion kinetics of pollutants through well-developed pore structures, thereby fully exposing the active adsorption sites. This paper systematically reviews the latest progress in lignin-based hydrogels for water treatment and discusses in depth the underlying logic of “structure construction–micromorphology–adsorption performance.” First, this review summarizes synthesis strategies ranging from molecular-level modification to morphology regulation, including nano-reinforcement, magnetic functionalization, and interpenetrating polymer networks. It then provides a pollutant-specific analysis of the adsorption mechanisms of lignin-based adsorbents. For heavy metal ions, such as Pb^2+^ and Cr(VI), removal is mainly associated with coordination/complexation, ion exchange, and redox reactions. For typical organic dyes, adsorption is primarily driven by π–π interactions, hydrogen bonding, and electrostatic attraction. The effects of environmental factors, such as pH, are also systematically discussed. Finally, considering current challenges related to mechanical strength, regeneration performance, and practical application, this review outlines future research directions for the development of multifunctional, integrated, and stimuli-responsive lignin-based adsorbents.

## 1. Introduction

With the acceleration of industrialization, heavy metal ions and organic dyes discharged from industries such as electroplating, mineral processing, and dyeing have accumulated extensively in water bodies, posing severe challenges due to combined pollution [1,2,3]. Heavy metal ions, due to their persistence, bioaccumulation, and strong mobility, can endanger the human central nervous system and organ health through biomagnification, even at extremely low concentrations [4,5,6,7]. Organic dyes, with their complex aromatic ring structures, not only significantly reduce water transparency and deteriorate dissolved oxygen levels, but their metabolites also carry potential risks of “three carcinogenic effects” (carcinogenicity, teratogenicity, and mutagenicity) [8,9,10,11,12]. Given their distinct chemical properties, heavy metal ions mostly exist in ionic forms and participate in complexation reactions, while organic dyes rely on hydrophobic and electrostatic interactions for migration. Conventional methods struggle to achieve simultaneous, efficient removal. Therefore, the development of an integrated remediation technology that combines broad-spectrum adsorption with rapid mass-transfer kinetics has become an urgent task in environmental engineering.

Among various remediation strategies, chemical precipitation, membrane separation, and advanced oxidation processes—although each exhibits distinct characteristics—respectively encounter engineering limitations, including high reagent consumption, severe membrane flux decline, and elevated operational energy consumption. In contrast, adsorption technology, due to its flexible process and low energy consumption, is regarded as one of the most promising technologies for widespread application [13,14,15,16]. From a sustainable development perspective, lignin-based renewable biomass materials offer unique advantages [17,18,19,20]. As the second most abundant natural aromatic polymer after cellulose, lignin features a dense distribution of phenolic hydroxyl groups, carboxyl groups, and benzene rings on its backbone. These structural moieties not only endow lignin with outstanding chemical reactivity but also provide an inherently favorable chemical foundation for the targeted capture of heavy metal ions and organic pollutants [21,22,23,24,25].

However, the complex molecular structure of lignin inevitably leads to limited solubility, pronounced steric hindrance, and inherent brittleness, which can cause issues such as powdering and structural loss during preparation and use. To address these drawbacks, transforming lignin into lignin-based hydrogels has been proven to be an effective strategy [26,27,28,29]. Specifically, the formation of a three-dimensional cross-linked network not only alleviates the physical defects of native lignin but also creates a well-developed porous architecture, establishing an interconnected 3D through-mass-transfer pathway that facilitates the diffusion of pollutants within the material matrix [30,31,32]. Nevertheless, network formation alone is insufficient to fully unlock lignin’s adsorption potential. The key challenge is that adsorption capacity and kinetic efficiency are often constrained by insufficient accessibility to functional groups and a limited density of effective binding sites. Consequently, recent advances have primarily enhanced lignin hydrogel adsorption performance from two complementary directions: (1) micro-molecular engineering, e.g., increasing the density and affinity of adsorption sites through sulfonation, fractionation/extraction, or graft copolymerization [33,34,35]; and (2) macro-structural construction, e.g., introducing nanofiber reinforcement or magnetic-responsive components or building semi-interpenetrating/interpenetrating polymer networks (IPNs) to realize synergistic improvements in adsorption capacity, mechanical stability, and recyclability [36,37,38].

Despite these substantial research achievements, existing reviews still tend to summarize reported studies rather than provide a critical and structured synthesis that consistently links structure construction, micro-/nano-morphology, and adsorption performance. Moreover, in many recent studies and related reviews, adsorption performance is frequently interpreted in a single-target manner, for example by focusing on only one specific heavy metal ion or one particular dye. This limits a broader understanding of how lignin hydrogel structures can be interpreted and compared across representative pollutant categories. As a result, mechanistic discussions often remain fragmented, without sufficiently adopting a classification-based perspective to connect interaction modes and design principles with different adsorption targets, particularly organic dyes and heavy metal ions.

To address this limitation, the novelty of this work lies in providing a category-oriented synthesis of lignin-based hydrogel adsorbents, rather than claiming full coverage of all pollutant systems or providing a dedicated review of mixed dye–metal co-contaminant systems. On this basis, this paper reviews recent progress in lignin-based hydrogels and analyzes their adsorption behavior toward two representative pollutant categories: organic dyes and heavy metal ions. The adsorption mechanisms relevant to these target categories are discussed, including coordination complexation, ion exchange, redox reactions, π–π interactions, hydrogen bonding, and electrostatic attraction. In addition, this review clarifies how key environmental factors, such as pH and ionic strength, modulate adsorption behavior for these representative pollutants. Finally, considering the limitations encountered in practical engineering applications, issues such as competitive/cooperative adsorption, natural organic matter, ionic strength, real wastewater matrices, regeneration, and scalability are discussed as future research needs for multifunctional and stimuli-responsive lignin-based hydrogels.

## 2. Data Collection and Methodology

To ensure a comprehensive and rigorous review of current advancements, a systematic literature search strategy was employed. The primary databases used for literature retrieval were Web of Science and Google Scholar. The search was conducted using a combination of exact keywords and relevant material identifiers, including “lignin-based hydrogels,” “heavy metal ions,” “organic dyes,” “synergistic removal,” “wastewater treatment,” and specific target ions such as Pb, Cr, Cu, and Cd, together with related descriptors (e.g., dyes).

To maintain the timeliness and relevance of this review, the collected literature mainly focused on publications from the past five years. During the screening process, greater priority was given to studies that (i) provided detailed mechanistic insights into the adsorption/removal process, (ii) assessed the regeneration and reuse capability of lignin-based hydrogels, and (iii) evaluated their practical performance in real or complex wastewater systems (rather than only using simplified laboratory aqueous solutions). Date: 27 March 2026.

## 3. Preparation Strategies of Lignin-Based Hydrogels and Their Adsorption Structure–Activity Relationships

**Figure 1:**
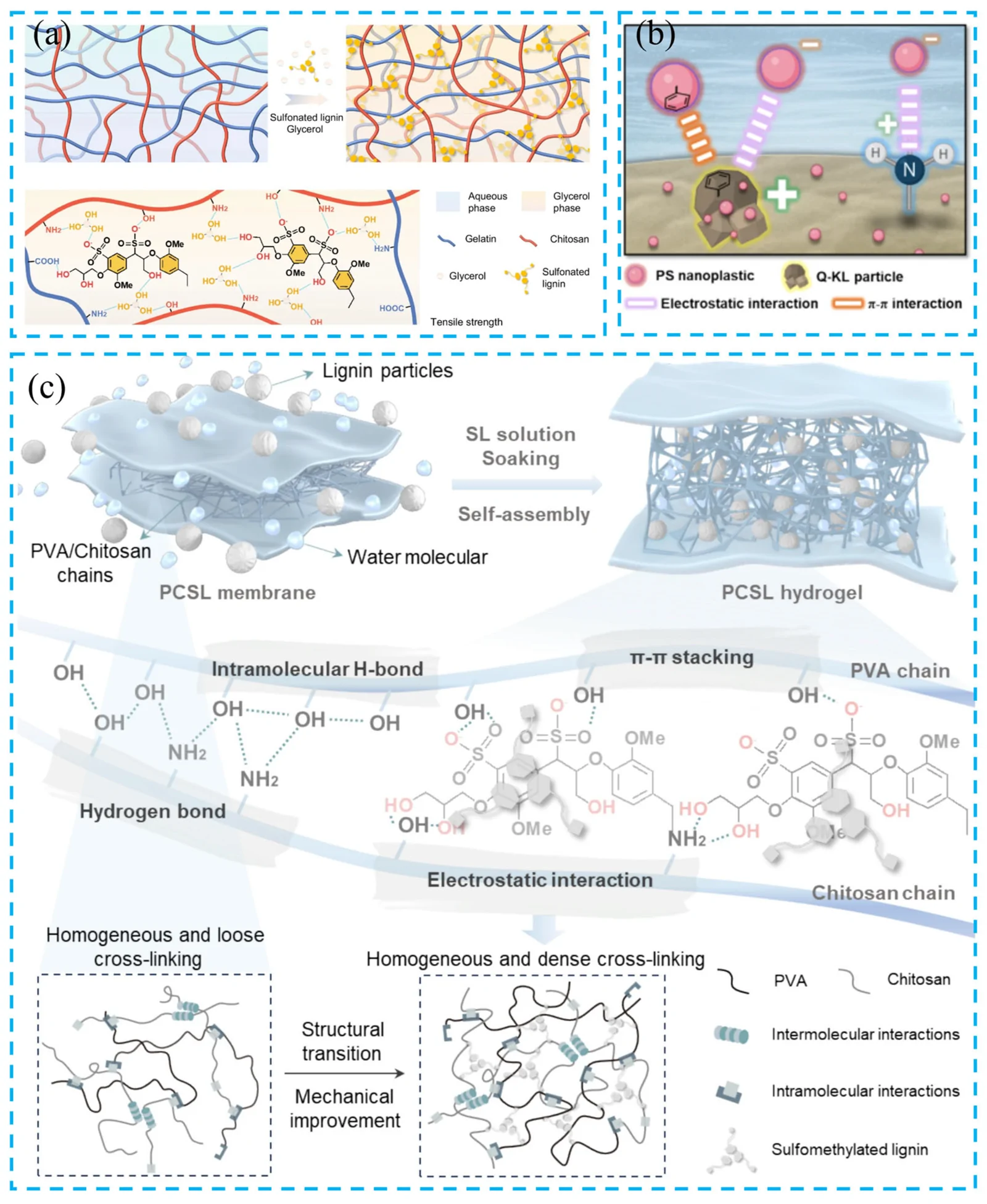
(**a**) Mechanism of non-covalent cooperative enhancement in CGSD bio-gel; reproduced with permission from reference [39], Copyright 2025, Springer Nature. (**b**) Schematic diagram of the mechanism for PS adsorption of CQ1; reproduced with permission from reference [40], Copyright 2025, Springer Nature. (**c**) Schematic diagram of the mechanism of PS nanoplastics adsorbing CQ1; reproduced with permission from reference [41], Copyright 2026, Elsevier.

Lignin, with its inherent aromatic backbone and abundant active functional groups, has emerged as an ideal precursor for constructing high-performance adsorptive hydrogels. The selection of cross-linking strategies during preparation directly dictates the material’s network topology and macroscopic adsorption performance. Among these strategies, chemical cross-linking constructs irreversible covalent networks via radical copolymerization or nucleophilic substitution. This endows the material with exceptional rigidity and chemical stability, enabling it to withstand complex wastewater environments. However, this approach must be carefully controlled to avoid steric hindrance and active-site masking effects caused by high cross-linking densities, which can restrict the mass-transfer of large adsorbates like macromolecular dyes (Figure 1a) [39]. In contrast, physical cross-linking enables green, mild preparation via non-covalent interactions such as hydrogen bonding, ionic complexation, and π–π stacking. The resulting flexible, highly swollen networks significantly promote the rapid diffusion and capture of heavy metal ions, though they often exhibit limited mechanical stability under harsh conditions (Figure 1b) [40]. To balance network strength with dynamic variability, dynamic reversible covalent cross-linking (e.g., Schiff base, borate ester, and Diels–Alder reactions) has been integrated into lignin hydrogel systems. This confers “smart” attributes such as self-healing, environmental stimuli responsiveness, and on-demand desorption/regeneration, thereby markedly extending the cyclic service life of the adsorbents in practical applications (Figure 1c) [41]. Furthermore, interpenetrating polymer networks (IPNs), formed by the topological interpenetration and physical entanglement of multi-component macromolecules, effectively overcome the brittleness inherent in traditional single-component lignin gels. The hierarchical porous structures constructed by IPNs achieve a synergy of rigidity and flexibility while maximizing the accessibility of lignin’s functional groups. Consequently, this architecture significantly enhances both the mass transfer kinetics and the overall adsorption capacity of heavy metal ions and large-volume organic dyes [42].

Lignin-based hydrogels offer a compelling balance of performance, cost, and sustainability, primarily achieved through composite formation and chemical modification. Their key advantages are threefold. First, using lignin, an inexpensive, renewable industrial by-product, as a base material and compounding it with biopolymers (e.g., chitosan, alginate) or inorganic nanomaterials (e.g., montmorillonite, MOFs) synergistically enhances mechanical strength, three-dimensional network stability, and adsorption capacity (e.g., Pb(II) uptake can increase from tens to several hundred, even over 700 mg/g). Second, chemical modifications such as amination or grafting allow for the precise introduction of high-density functional groups, granting the material high selectivity for specific pollutants (e.g., anionic dyes and heavy metal ions), pH responsiveness, and easy regeneration. Finally, the main components exhibit good biocompatibility and potential biodegradability, aligning with green chemistry principles. However, notable drawbacks exist. Compared to high-performance activated carbon or synthetic resins, their specific surface area and adsorption kinetics are often lower. The long-term swelling stability and mechanical durability of the network, especially under extreme pH or high-ionic-strength conditions, require further improvement. Moreover, while the raw material is cheap, complex modification or composite processes can increase overall production costs. Therefore, lignin-based hydrogels are not designed for ultimate performance but represent a specialized class of adsorbents tailored for efficient, green, and cost-effective wastewater treatment in specific application scenarios.

## 4. Adsorption Mechanism of Lignin-Based Composite Hydrogels

The removal of organic dyes and heavy metal ions by lignin-based hydrogels is a complex process involving the synergistic coupling of physical adsorption and chemical trapping. Electrostatic interactions serve as the primary driving force, enabling rapid capture via Coulombic attraction between abundant oxygen-containing groups on the lignin surface and ions present in aqueous solutions. Its efficiency is significantly regulated by pH and surface charge modification (e.g., amination). For instance, Wei et al. [43] introduced acryloyloxyethyltrimethyl ammonium chloride (DAC) via free radical graft copolymerization to construct a ternary copolymer hydrogel (LAD). The adsorption behavior follows the Langmuir isotherm (monolayer adsorption) and the pseudo-second-order kinetic model. Thermodynamic analysis confirms that this process is spontaneous, exothermic, and entropy-reducing. Under the synergistic effect of electrostatic interactions and hydrogen bonding, the AR73 removal rate by this hydrogel reaches 95.18%, and the maximum adsorption capacity reaches 409.84 mg/g. For transition-metal ions, electron-rich atoms (such as oxygen and nitrogen) in lignin form highly stable chelating complexes via coordination, resulting in strong binding and excellent selectivity.

Meanwhile, the unique phenylpropane skeleton of lignin endows the material with significant π–π stacking and hydrophobic interactions, which have a decisive advantage in capturing organic dye molecules with complex conjugated structures. The chitosan–lignin (CL) composite microspheres developed by Bashir et al. [44] achieved a removal rate of 96% for DB-218 at pH 4 and a maximum adsorption capacity of 93.5 mg/g, which is significantly better than single-component materials, through the synergistic effect between protonated amino groups of chitosan and hydroxyl and carbonyl groups of lignin. In addition, the widely distributed hydrogen-bonding network plays an auxiliary anchoring role in the initial enrichment of pollutants at the interface and maintains structural stability, while the well-developed three-dimensional pore network eliminates mass-transfer resistance and enhances the spatial accessibility of active sites, thereby improving overall adsorption capacity and the kinetic rate.

Multiple interfacial behaviors jointly determine adsorption efficiency. Electrical double layer interaction: zeta potential tests confirm the pH-dependent surface charge of lignin hydrogels, dominating electrostatic attraction/repulsion between adsorbent and ionic pollutants. Interfacial hydrogen bonding and π–π stacking: aromatic skeletons and hydroxyl groups on lignin form instantaneous interfacial binding layers to capture dye molecules. Interfacial mass-transfer boundary layer: the hierarchical pore structure of IPN hydrogels thins the boundary layer and reduces diffusion resistance. Competitive interfacial adsorption: coexisting inorganic ions occupy surface active sites and inhibit target pollutant capture. Combined with XPS, zeta potential, and CLSM characterization results, we elaborate on how interfacial microscopic interactions control macroscopic adsorption capacity and selectivity.

## 5. Application of Lignin-Based Hydrogels in Wastewater Treatment

### 5.1. Adsorptive Removal of Organic Pollutants from Wastewater Using Lignin-Based Hydrogels

#### 5.1.1. Cationic Dyes

Lignin-based hydrogels, as an emerging class of biomass-derived adsorbents, show promising potential for treating cationic dye wastewater. Their core advantages stem from the abundant active functional groups in the lignin molecular structure, such as phenolic hydroxyl groups and carboxyl groups. These groups not only serve as cross-linking reaction sites to construct a three-dimensional network skeleton but also enable efficient capture of dye molecules through multiple interaction mechanisms, including electrostatic attraction, hydrogen bonding, and π–π stacking.

**Figure 2:**
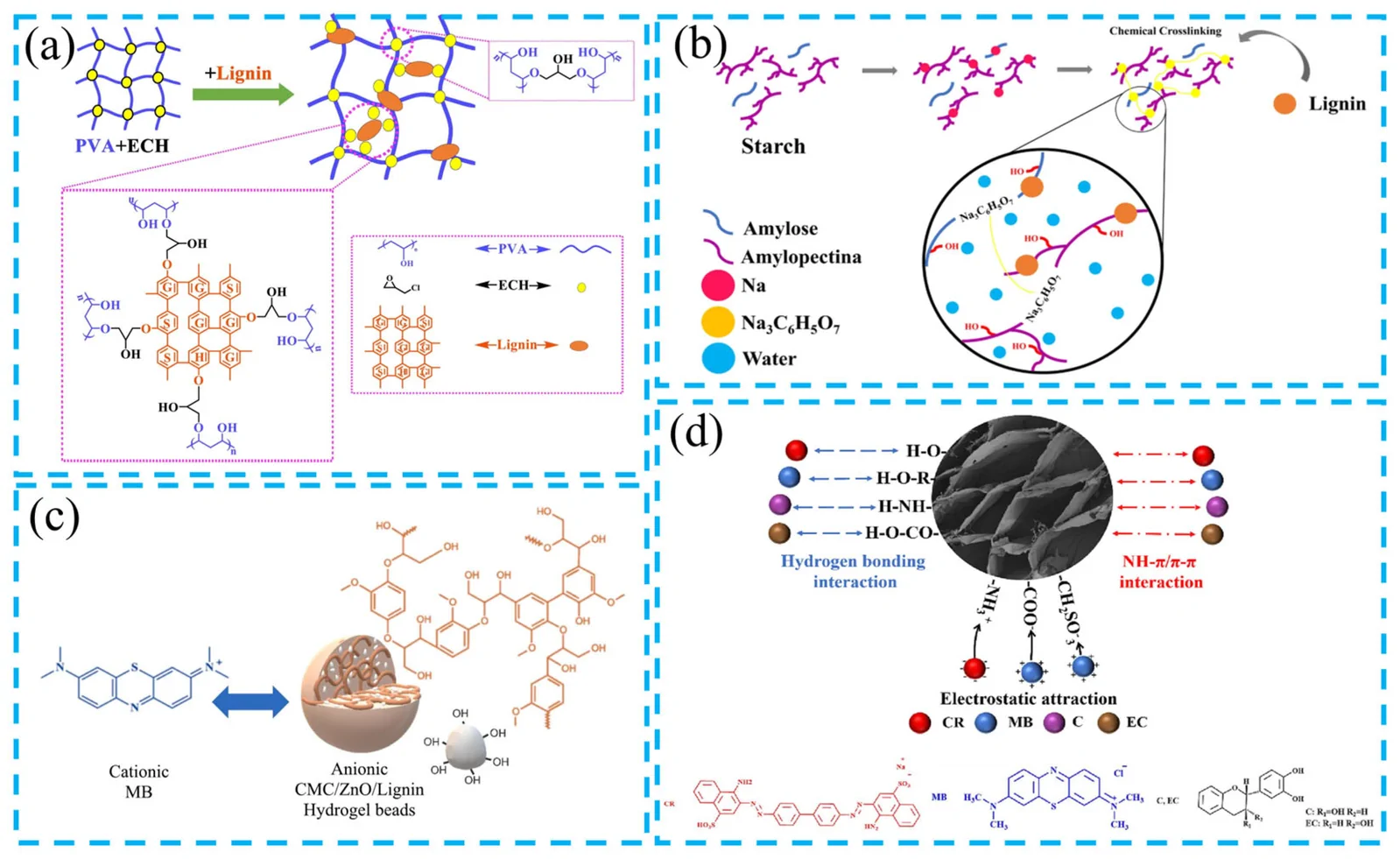
(**a**) Structural mechanism of lignin–polyvinyl alcohol hydrogel; reproduced with permission from reference [47], Copyright 2019, Elsevier. (**b**) Citric acid-initiated chemical cross-linking process in alkaline medium and the adsorption mechanism of lignin in the hydrogel matrix; reproduced with permission from reference [50], Copyright 2024, Springer Nature. (**c**) Adsorption mechanism of cationic MB onto anionic CMC/ZnO/lignin hydrogel beads; reproduced with permission from reference [52], Copyright 2024, Elsevier. (**d**) Possible adsorption mechanisms of CR, MB, C and EC on CS-LIG aerogel; reproduced with permission from reference [53], Copyright 2023, Elsevier.

In terms of material construction strategies, researchers first focus on balancing the structural stability and adsorption performance of hydrogels by optimizing cross-linking systems (Table 1). In single chemical cross-linking pathways, epoxy cross-linking agents (such as PEGDE and ECH) are widely used due to their high reactivity with lignin’s hydroxyl groups. Yu et al. [45] prepared lignosulfonate-g-acrylic acid (LS-g-AA) hydrogels through laccase/t-BHP-initiated grafting of acrylic acid onto lignosulfonate, with MBA used as the cross-linker. FTIR spectra confirmed the successful grafting of acrylic acid and the formation of a cross-linked hydrogel network. The introduced carboxyl groups contributed to efficient methylene blue (MB) adsorption, with a maximum capacity of 2013 mg/g. The adsorption process fitted the Freundlich isotherm and pseudo-second-order kinetic model. Nevertheless, the desorption efficiency was only about 50%, suggesting limited recyclability. To address this issue, Jung et al. [46] proposed a “double reinforcement” strategy that combines kraft lignin (KL) with polyvinyl alcohol (PVA). FTIR and SEM were used to verify the synergistic cross-linking/structural evolution: specifically, ECH-mediated covalent bonding provides chemical stabilization, while freeze–thaw cycles induce physical entanglement that strengthens the network. During adsorption, KL particles not only act as a structural reinforcing phase to improve compression performance but also synergistically capture dye molecules in a targeted manner through multiple mechanisms, including electrostatic interactions, hydrogen bonding, and π–π stacking. The adsorption capacities of this composite system for methylene blue (MB) and CV are greatly increased to 193.8 mg/g and 190.0 mg/g, respectively, and the adsorption efficiency remains above 86% after 10 cycles. Based on cross-linking network optimization, the diversity of lignin raw material sources offers ample scope for customized regulation of material properties. Wu et al. [47] systematically evaluated the effects of alkali lignin, softwood/hardwood lignin, and agricultural and forestry waste lignin on the properties of Lignin–PVA superabsorbent hydrogels. Dynamic strain sweep and FTIR were used to connect “lignin source, network structure/interaction ability, and macroscopic swelling/adsorption.” The specific molecular weight distribution and abundant phenolic hydroxyl groups of lignin from different sources were shown to play significant roles in structural reinforcement and hydrophilization in the gel network, effectively overcoming the poor mechanical and swelling performance of traditional PVA hydrogels. The incorporation of lignin increases the swelling ratio of the gel from 92 g/g to a maximum of 465 g/g under weakly acidic conditions. It exhibits excellent adsorption capacities of 196 mg/g, 169 mg/g, and 179 mg/g for rhodamine 6G, crystal violet, and ferrocene, respectively. Overall, these results demonstrate that tailoring the lignin feedstock can rationally regulate the microscopic topology and the density of adsorption-active sites, thereby dictating adsorption behavior (Figure 2a).

With increasing demand for green materials and convenient recycling, research has shifted to initiator-free physical cross-linking and magnetic directional separation. Huang et al. [48] avoided the environmental risks posed by traditional chemical cross-linking agents. SEM, SEM-EDS, XRD, FTIR, and AFM images were used to verify that a fully physically blended network was successfully constructed and to show how inorganic/biopolymer components contribute to the internal structure. The incorporation of LCN not only significantly increased the storage modulus of the system by 163.0% and endowed the material with excellent structural support, but also enabled efficient removal of methylene blue (MB) (>95.7%) while preserving the material’s eco-friendliness to the greatest extent. This structure–property relationship links the enhanced mechanical integrity (facilitating mass transfer and site accessibility) with the improved adsorption performance. To address the engineering bottleneck of traditional adsorbents’ slow recovery in large-scale water bodies, Meng et al. [49] developed lignin-derived magnetic hydrogel microspheres (LDMHMs). FTIR and SEM were used to confirm lignin amination and the successful formation of composite particles (lignin/cellulose/Fe_3_O_4_), which collectively introduce abundant adsorption functionalities and magnetic responsiveness. In this study, diethylenetriamine (DETA) was used to modify lignin via amination, which introduced abundant, highly active adsorption sites; the modified lignin was then compounded with cellulose and Fe_3_O_4_ nanoparticles to prepare particles. This material not only shows extremely high affinity for organic dyes such as malachite green, methylene blue and Methyl Orange (with equilibrium adsorption capacities reaching 155 mg/g, 43 mg/g, and 39 mg/g respectively), but also exhibits excellent co-adsorption removal capacity for heavy metal ions such as Hg^2+^ (55 mg/g), Pb^2+^ (33 mg/g) and Ni^2+^ (23 mg/g). More importantly, for practical engineering, the magnetic microspheres can be easily desorbed under mild acidic elution conditions, and the adsorption capacity retention remains above 90% after multiple cycles, enabling an effective combination of high adsorption capacity, broad-spectrum removal, and efficient separation and regeneration.

To further broaden the scope of application, researchers have begun to explore the blending modification of lignin with multi-component matrices to address the complex industrial wastewater environment (Table 1). Fernandes Pereira et al. [50] developed an eco-friendly starch-based composite hydrogel containing lignin, using starch as the matrix, sugarcane bagasse as the raw material, and trisodium citrate as a green cross-linking agent. SEM, XPS, TGA, and FTIR were used to support the successful incorporation/cross-linking of components and to evidence the charge-state evolution responsible for adsorption selectivity. This system exhibited unique charge-switching properties: it could remove chromate anions under acidic conditions and demonstrated extremely high removal efficiency (exceeding 90%) for the cationic dye methylene blue (MB) under weakly alkaline conditions, with an adsorption capacity reaching 99.4 mg/g after 70 min. The experimental data were highly consistent with the Freundlich isotherm model, indicating that the adsorption process was thermodynamically favorable (Figure 2b). Concurrently, to tackle the persistent issue of lignin leaching from matrices, Yeasmin et al. [51] covalently embedded lignin into a polyvinyl alcohol (PVA) matrix via direct esterification using polyamide-amine epichlorohydrin (PAE), effectively resolving the lignin leaching problem. ATR-FTIR, SEM, and TGA (including FTIR re-evidence of chemical bonding) were used to confirm the covalent immobilization and improved structural stability. They developed a highly efficient and eco-friendly composite hydrogel for MB removal from water using lignin derived from Ayous wood waste. Characterization results revealed that lignin from different sources significantly influenced hydrogel performance. Compared with commercial lignin (64% removal rate) and unmodified hydrogel (9%), the hydrogel prepared from Ayous wood waste lignin exhibited the highest adsorption performance, achieving an exceptionally high MB removal rate of 88%. This study provides a typical paradigm for the high-value utilization of industrial waste.

Currently, the development of multifunctional composite systems represents the frontier of this field, transitioning from mere “physical capture” to “integration of adsorption and transformation” and “precision separation.” Fu et al. [52] addressed the issues of weak chemical interactions and poor reusability in traditional carboxymethyl cellulose (CMC) hydrogels by incorporating CMC with ZnO nanoparticles. SEM and FTIR were used to demonstrate the porous/functionalized network formation and the abundant oxygen-containing groups introduced by lignin, which together provide adsorption binding sites. The incorporation of lignin facilitated the formation of a porous structure and introduced numerous hydroxyl and carboxyl groups (Figure 2c). ZnO increased the surface roughness, and the synergistic effect of these components enhanced the MB adsorption capacity to 276.79 mg/g. Notably, MB photodegradation was achieved via ZnO excitation under ultraviolet light, with in situ regeneration of active sites, thereby realizing the dual function of “adsorption–photocatalytic self-cleaning.” Zhu et al. [53] synthesized a novel chitosan–DES lignin composite aerogel (CSLIG aerogel) through solution mixing and freeze-drying with copper plates, using deep eutectic solvent (DES)-extracted lignin from grapevines and chitosan. SEM, XPS, TGA, FTIR and XRD were used to support the microstructure/chemical bonding and stability, linking the lignin-related functional groups to the observed adsorption selectivity and recyclability. This system relies on the strong π–π interactions and hydrogen-bonding networks provided by the abundant aromatic and nucleophilic groups of lignin to capture toxic dyes. The material exhibited excellent and recyclable adsorption performance, with adsorption capacities of up to 175.84 mg/g for Congo red and 95.68 mg/g for methylene blue (with only a 1.3% performance degradation after four cycles). The removal rates for catechin and epicatechin reached 86.42% and 90.85%, respectively. These findings indicate that research on lignin-based materials is advancing toward deeper areas, such as biorefining and resource recycling (Figure 2d).

#### 5.1.2. Anionic Dyes

In the treatment of anionic dyes using lignin-based hydrogels, the core challenge is overcoming electrostatic repulsion between natural lignin and anionic dyes. Therefore, introducing cationic groups via chemical modification (such as amination and quaternization) to alter the surface charge is a key strategy for improving the adsorption performance of lignin-based hydrogels.

Introducing high-charge-density cationic groups (such as amino groups and quaternary ammonium groups) to achieve surface charge conversion is a prerequisite strategy for overcoming electrostatic repulsion. Wang et al. [54] used black-liquor lignin modified by 2,3-epoxypropyltrimethylammonium chloride and acryloyl chloride (namely cationic lignin acrylate, CKLA) to synthesize cationic lignin (CKLA) hydrogels via polymerization with acrylamide and cross-linking agents (such as N, N′-Methylenebisacrylamide). Guided by ^31^P NMR, SEM, and FTIR analyses, the successful grafting of cationic groups and the formation of a porous cross-linked network were confirmed. This structural evolution effectively addresses the limited number of active sites in natural lignin, enabling the material to exhibit efficient, stable adsorption performance. When 5 mg of the hydrogel containing 20% lignin was used to treat a dye solution with an initial concentration of 50 mg/L and a pH of 7, the maximum removal rate efficiency reached 95%. Meanwhile, the hydrogel exhibits excellent reusability, with removal efficiency remaining above 80% after five adsorption cycles. To address the fact that industrial printing and dyeing wastewater is often strongly alkaline, Díaz et al. [55] synthesized a novel P(ClAPTA-AL) biocomposite hydrogel via free radical polymerization, using aminated lignin (AL) with amino groups introduced via the Mannich reaction and the synthetic monomer (3-acrylamidopropyl) trimethylammonium chloride (ClAPTA). Through SEM, EDX, and FTIR characterizations, the successful incorporation of aminated lignin and the uniform distribution of elements within the polymer matrix were verified, providing a mechanistic basis for why the material exhibits excellent structural integrity and thermal stability, and shows an adsorption capacity of 102.1 mg/g for Alizarin Red S under optimal conditions (pH 12.0, 20 °C, contact time 120 min). Moreover, it exhibits extremely high structural stability and reusability: the adsorption efficiency remains approximately 99% after four cycles and 81.1% after seven cycles, highlighting the material’s structural stability under extreme pH conditions.

The composite design further optimizes the pore structure and the synergistic adsorption effect of hydrogels by introducing additional biological macromolecules. In the biomass composite system, Li et al. [56] constructed a lignin/cellulose hydrogel with a three-dimensional network structure via the self-assembly of lignosulfonate and cellulose. Comprehensive characterizations using SEM-EDS, XPS, TGA, FTIR, and XRD elucidated the self-assembly mechanism driven by non-covalent interactions and confirmed the thermal stability of the composite. These techniques revealed that the abundant hierarchical pore structure of this material significantly improves its water absorption and retention properties and exhibits excellent adsorption capacity for macromolecular organic dyes. For the anionic dye Congo red (CR), the optimal adsorption performance is achieved at pH 6 and 45 °C, and the adsorption process conforms to the Langmuir isothermal model (monolayer adsorption) and the pseudo-second-order kinetic model, with a maximum adsorption capacity of 294.0 mg/g; the preparation process is green and straightforward. Notably, the “waste treating waste” strategy proposed by Diksha et al. [57] is particularly outstanding: supported by FESEM/EDS, FTIR, and XRD evidence, the successful cross-linking of black liquor components (lignin/hemicellulose) with sodium alginate was proven to form a robust network. The BL/SA composite hydrogel prepared using black liquor (rich in lignin and hemicellulose) and sodium alginate achieves a maximum adsorption capacity of 650 mg/g at a dye concentration of 1500 mg/L, with a decolorization rate of up to 95.54% for copper phthalocyanine blue dyes within 12 min, while also exhibiting excellent recyclability (can be used continuously for four cycles).

**Figure 3:**
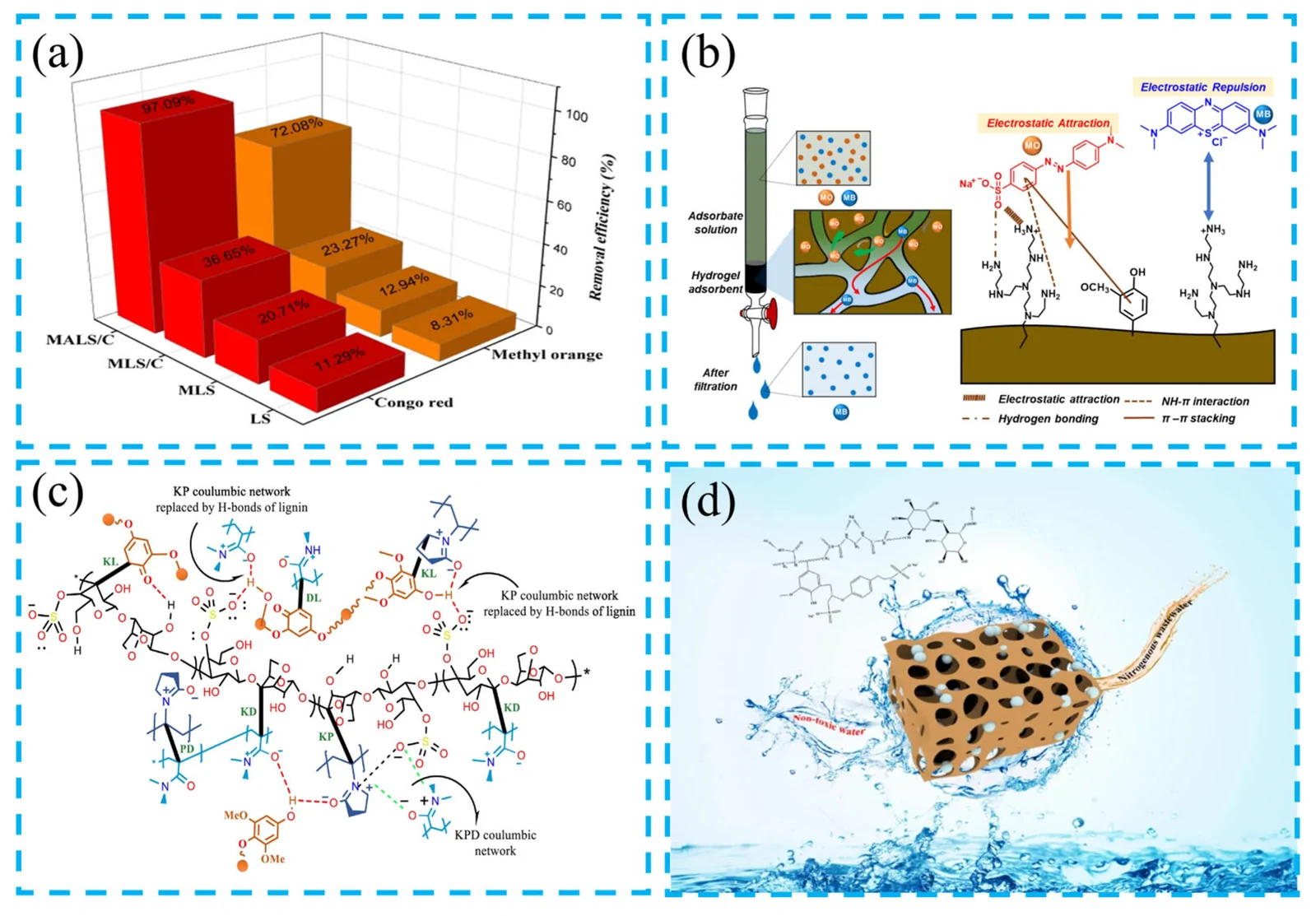
(**a**) Adsorption of Congo red and Methyl Orange on LS, MLS, MLS/C, and MALS/C; reproduced with permission from reference [58], Copyright 2022, Springer Nature. (**b**) Hypothetical selective adsorption mechanism of MB and MO on BALH; reproduced with permission from reference [59], Copyright 2024, Elsevier. (**c**) Schematic diagram of KPLD hydrogel network, where thick lines represent cross-linking between trehalose (K), PVP (P), lignin (L), and pDMA (D), as well as Coulombic interactions between K-P-D, and H-bond substitution of lignin; reproduced with permission from reference [60], Copyright 2025, Elsevier. (**d**) New lactose–lignin hydrogel for wastewater treatment; reproduced with permission from reference [62], Copyright 2024, Springer Nature.

To address the difficulty of adsorbent recovery and further increase the upper limit of adsorption capacity, magnetic functionalization and dendrimer modification have emerged as breakthrough directions. Borsalani et al. [58] successfully developed a magnetic aminated lignosulfonate/carbon (MALS/C) composite adsorbent using lignosulfonate, a waste product from the paper industry. A combination of FESEM, SEM-EDS, TGA, FTIR, and XRD confirmed the successful amination and carbon composite structure, while VSM analysis verified its magnetic responsiveness. Consequently, MALS/C exhibits excellent paramagnetic recovery performance for Congo red (CR) and Methyl Orange (MO), with maximum removal rates of 97.09% and 72.08%, respectively, and maximum adsorption capacities of 167.61 mg/g and 121.28 mg/g, respectively. Studies have shown that the adsorption process is a spontaneous endothermic reaction, and the kinetic and thermodynamic data follow the pseudo-second-order kinetic model and the Langmuir monolayer adsorption model, respectively. The adsorbent can maintain a high removal efficiency after five regeneration cycles (Figure 3a). In terms of exploring the adsorption capacity limit, Heo et al. [59] prepared a lignin-based hydrogel (BALH) with a dendritic activated network and ultrahigh active site density through an epoxy ring-opening reaction using lignin, dendritic polyethyleneimine (PEI), and polyethylene glycol diglycidyl ether (PEGDE). The successful ring-opening grafting and the formation of a highly dense active-site network were systematically verified by ^13^C NMR, SEM-EDS, XPS, FTIR, and XRD. This structural proof explains why, due to the positively charged surface imparted by PEI, the adsorbent selectively and efficiently captures anionic dyes (such as Methyl Orange, MO) via strong electrostatic interactions. Experimental results show that the adsorption capacity of BALH for MO reaches approximately 900–930 mg/g, and the process follows pseudo-second-order kinetics and the Langmuir isotherm model, indicating spontaneous endothermic chemisorption. In addition, the material exhibits outstanding regeneration performance and maintains extremely high removal efficiency after 10 adsorption–desorption cycles (Figure 3b).

The design of dual-component composite hydrogels represents a cutting-edge direction that has evolved from “single-mode adsorption” to “broad-spectrum adsorption”. Kubra et al. [60] overcame the limitations of traditional chemical cross-linking and constructed a quaternary composite network (carrageenan/PVP/lignin/DMA) with precisely controlled composition using γ-ray radiation-induced technology. Microstructural and spectroscopic evidence from SEM, FTIR, and XRD confirmed the successful integration of the four components without phase separation. This system utilizes the sulfate-mediated amide activation mechanism to deeply integrate the hydrogen-bonding network of lignin with multi-component ionic interactions, endowing the material with excellent charge-neutralization capability and enabling synchronous, synergistic capture of the cationic dye (MB) and the anionic dye (CR). The maximum adsorption capacities reach 405 mg/g and 417 mg/g, respectively (Figure 3c). In addition, the hydrogel exhibits good cycling stability (reusable for at least four cycles).

Importantly, this study introduced ecotoxicological evaluation, confirming the biosafety of the material after wastewater treatment and its potential fertilizer effect on crop growth, providing a new perspective for the terminal disposal of biomass hydrogels. Unlike the radiation-induced physical/chemical hybrid cross-linking strategy, Kim et al. [61] focused on constructing functional networks via precise chemical modification. Using XPS and FTIR to monitor the evolution of surface chemical states and functional groups, they confirmed that a cross-linked aminated lignin hydrogel (CALH) was successfully synthesized via the Mannich reaction using PEGDGE as the cross-linker. Benefiting from the introduction of high-density amino functional groups, CALH not only strengthens electrostatic attraction but also constructs multi-dimensional adsorption sites through NH–π interactions. The experimental adsorption results show that the maximum adsorption capacities of the hydrogel for CR and MO reach 170.42 mg/g and 195.45 mg/g, respectively. Although its maximum adsorption capacity (170.42–195.45 mg/g) is slightly lower than that of the radiation cross-linking system, it exhibits extremely high structural stability and chemical affinity for the targeted decolorization of industrial dye wastewater.

Finally, the functional transition from “adsorption–separation” to “catalytic-degradation” has opened up a new pathway for the applications of lignin-based hydrogels. Zhang et al. [62] synthesized a novel composite hydrogel with self-reduction capability via self-assembly using carboxylated lactose, sodium lignosulfonate, and polyacrylic acid as raw materials. Its well-developed porous network structure provides abundant anchoring sites for silver ions (Ag^+^), and the hydrogel’s inherent reduction ability enables the in situ conversion of Ag^+^ into silver nanoparticles, thereby creating highly active catalytic sites. This composite material exhibits excellent catalytic performance: it not only demonstrates good conversion stability under fixed-bed reaction conditions (conversion rate > 80% after 540 min) but also possesses strong environmental adaptability in both tap water and simulated seawater. After eight cycles, the catalytic efficiency remains as high as 99.7%, thus illustrating the functional transition from adsorption–separation to catalytic-degradation (Figure 3d).

In conclusion, environmental conditions (such as pH, temperature, and concentration) profoundly affect the adsorption performance of lignin-based hydrogels by regulating the interaction between the adsorbate and the adsorbent (especially electrostatic interaction) and the mass-transfer process. And through meticulous material design (such as cation modification, composite construction, and functionalization) to actively adapt and optimize the influence of these conditions, it is the key to enhancing its application efficiency in actual complex wastewater.

**Table 1:** Lignin-based hydrogel/composite adsorbents for dye remediation.

Material Design	Target Pollutant(s)	Max. Capacity	Characterizations	Key Mechanisms	Ref.
Kraft lignin + PVA + ECH (double reinforcement)	MB, CV	MB: 193.8 mg/g; CV: 190.0 mg/g	FTIR, SEM	Physical entanglement + chemical stabilization; >86% retention after 10 cycles.	[46]
Diverse lignins + PVA	Rhodamine 6G, CV, ferrocene	R6G: 196 mg/g; CV: 169 mg/g	Dynamic strain sweep, FTIR	Lignin source dictates topology; high swelling ratio (up to 465 g/g).	[47]
LCN (initiator-free physical blending)	Methylene blue (MB)	>95.7% removal	SEM, EDS, XRD, FTIR, AFM	Eco-friendly physical network; 163% increase in storage modulus.	[48]
LDMHMs (aminated lignin/cellulose/Fe_3_O_4_)	Malachite green, MB, MO, heavy metal ions	MG: 155 mg/g; MB: 43 mg/g	FTIR, SEM	Magnetic directional separation; broad-spectrum removal; >90% retention.	[49]
Starch + sugarcane bagasse lignin + citrate	MB, chromate anions	MB: 99.4 mg/g	SEM, XPS, TGA, FTIR	pH-dependent charge-switching properties for selective adsorption.	[50]
Ayous wood waste lignin + PVA + PAE	MB	88% removal	ATR-FTIR, SEM, TGA	Covalent embedding prevents lignin leaching; waste valorization.	[51]
CMC + ZnO + lignin	MB	276.79 mg/g	SEM, FTIR	Integration of adsorption and UV-excited photocatalytic self-cleaning.	[52]
Chitosan + DES-extracted lignin aerogel	Congo red (CR), MB, catechin	CR: 175.84 mg/g MB: 95.68 mg/g	SEM, XPS, TGA, FTIR, XRD	Strong π–π and H-bonding; biorefining concept; highly recyclable.	[53]
CKLA (cationic lignin) + acrylamide + MBA	Anionic dyes	95% removal	^31^P NMR, SEM, FTIR	Surface charge conversion via cationic groups overcomes electrostatic repulsion.	[54]
P(ClAPTA-AL) (aminated lignin + ClAPTA)	Alizarin Red S	102.1 mg/g	SEM, EDX, FTIR	High structural stability and reusability under extreme alkaline conditions (pH 12.0).	[55]
Lignosulfonate + cellulose	Congo red (CR)	294.0 mg/g	SEM-EDS, XPS, TGA, FTIR, XRD	Non-covalent self-assembly; hierarchical pore structure.	[56]
Black liquor + sodium alginate	Cu-phthalocyanine blue	650 mg/g (95.54%)	FESEM, EDS, FTIR, XRD	“Waste treating waste” strategy; ultra-fast decolorization (12 min).	[57]
MALS/C (magnetic aminated lignosulfonate/carbon)	CR, Methyl Orange (MO)	CR: 167.61 mg/g; MO: 121.28 mg/g	FESEM, EDS, TGA, FTIR, XRD, VSM	Magnetic recovery; spontaneous endothermic chemisorption.	[58]
BALH (lignin + dendritic PEI + PEGDE)	MO	900–930 mg/g	^13^C NMR, SEM-EDS, XPS, FTIR, XRD	Ultrahigh active site density via dendritic network; extreme capacity.	[59]
Carrageenan/PVP/lignin/DMA (γ-ray radiation)	MB (cationic), CR (anionic)	MB: 405 mg/g; CR: 417 mg/g	SEM, FTIR, XRD	Synchronous synergistic capture; includes ecotoxicological evaluation.	[60]
CALH (aminated lignin + PEGDGE)	CR, MO	CR: 170.42 mg/g; MO: 195.45 mg/g	XPS, FTIR	High-density amino groups enhance electrostatic and NH–π interactions.	[61]
Carboxylated lactose + lignosulfonate + PAA + Ag^+^	Dyes (catalytic degradation)	>80% conversion (540 min)	-	In situ reduction of Ag^+^ to Ag NPs; transition from adsorption to catalysis.	[62]

Note: PEGDE/PEGDGE (polyethylene glycol diglycidyl ether); PVA (polyvinyl alcohol); ECH (epichlorohydrin); LDMHMs (lignin-derived magnetic hydrogel microspheres); PAE (polyamide-amine epichlorohydrin); CMC (carboxymethyl cellulose); ZnO (zinc oxide); DES (deep eutectic solvent); CKLA (cationic lignin acrylate); MBA (*N,N*′-methylenebisacrylamide); ClAPTA ((3-acrylamidopropyl) trimethylammonium chloride); AL (aminated lignin); MALS/C (magnetic aminated lignosulfonate/carbon); BALH (dendritic activated lignin hydrogel); PEI (polyethyleneimine); PVP (polyvinylpyrrolidone); DMA (*N,N*-dimethylacrylamide); CALH (cross-linked aminated lignin hydrogel); PAA (polyacrylic acid); Ag NPs (silver nanoparticles); CV (crystal violet); MB (methylene blue); MO (Methyl Orange); CR (Congo red); R6G (rhodamine 6G).

### 5.2. Adsorptive Removal of Heavy Metal Ions from Wastewater by Lignin-Based Hydrogels

#### 5.2.1. Pb

Research on the application of lignin-based hydrogels for lead ion (Pb(II)) wastewater treatment demonstrates a systematic development trajectory from material design and mechanism analysis to engineering applications. The core lies in constructing an efficient complexation adsorption system through composite design and functional group optimization. Chemical complexation, especially coordination involving oxygen-, nitrogen-, and sulfur-containing groups, is the dominant mechanism for Pb(II) adsorption by lignin-based hydrogels.

To maximize such chemical complexation effects, researchers have developed a variety of efficient synthetic strategies (Table 2). Zhao et al. [63] utilized a simple one-pot Mannich reaction to prepare a novel chitosan/lignin hydrogel (CSL). Comprehensive characterizations using ^13^C NMR, SEM-EDS, XPS, and FTIR confirmed that this system successfully integrated hydroxyl groups, amino groups, and an ether-bond network, providing the structural basis that enables the rapid capture of metal ions. The adsorption equilibrium times for the CSL1 hydrogel with Pb(II) and Cu(II) are only 1 and 2 min, respectively, with maximum adsorption capacities of 139.86 mg/g and 98.71 mg/g, respectively. Thermodynamic parameters indicate that this Langmuir-type monolayer adsorption is spontaneous, endothermic, and entropy-increasing. Following the idea of polymer grafting, Sun et al. [64] grafted acid-hydrolyzed lignin onto the surface of a polyacrylic acid network (AHL-g-PAA). Structural and morphological evidence from SSNMR, SEM, FTIR, and XRD verified the successful grafting process and the formation of a cross-linked network. These analyses revealed that such modification not only endows the AHL-g-PAA hydrogel with pH-responsive sensitivity but also further increases the density of complexation sites, resulting in a maximum Pb(II) adsorption capacity of 235 mg/g (consistent with the pseudo-second-order and Langmuir models). In addition, physical field-assisted synthesis also exhibits unique advantages. Wang et al. [65] constructed a dense cellulose–lignin composite hydrogel (UCHy) under ultrasonic radiation. Spectroscopic analyses via FTIR and XPS confirmed the chemical interactions between cellulose and lignin, while also demonstrating how the ultrasonic cavitation effect promotes the formation of a continuous porous microstructure. The swelling kinetic characteristics of this non-Fickian diffusion (consistent with the Schott model) enable it to show excellent adaptability over a wide range of pH and temperature. The optimal adsorption capacity is as high as 786.16 mg/g (consistent with the Langmuir/Freundlich and pseudo-second-order models).

**Figure 4:**
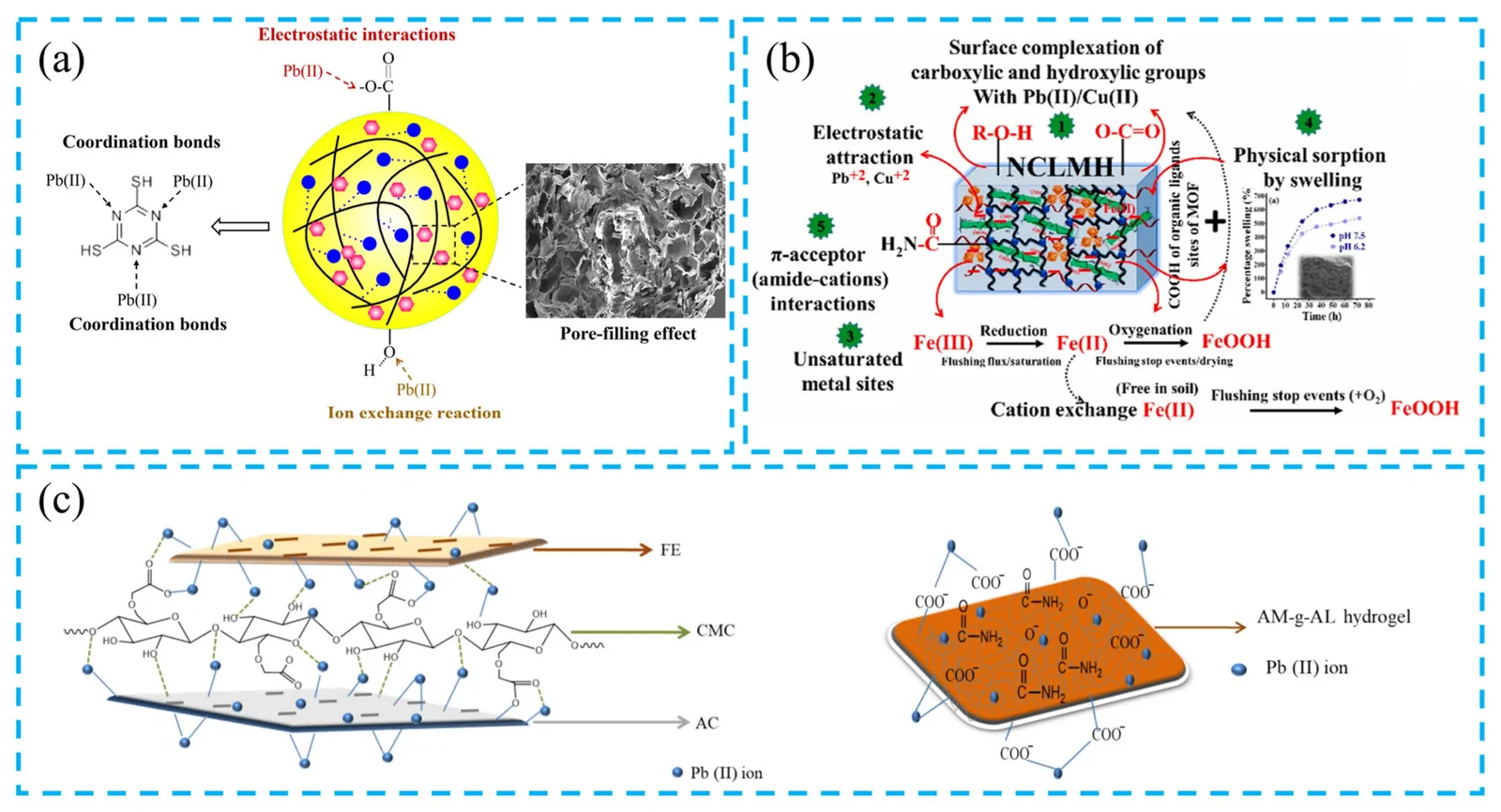
(**a**) Schematic diagram of the mechanism for Pb (II) adsorption by hydrogel-II; reproduced with permission from reference [67], Copyright 2024, Elsevier. (**b**) Mechanism for Pb and Cu removal induced by the NCLMH adsorbent; reproduced with permission from reference [69], Copyright 2024, Elsevier. (**c**) Possible adsorption mechanism of the FE-CMC-AC adsorbent; reproduced with permission from reference [71], Copyright 2025, Elsevier.

In terms of composite system construction, the incorporation of natural polymers such as sodium alginate (SA) not only enhances the mechanical strength of the gel but also facilitates the engineering transition of adsorption processes from static batch to dynamic continuous (fixed-bed) operation (Table 2). Zheng et al. [66] and Wang et al. [67] systematically investigated the hydrodynamic behavior of lignin/SA composite hydrogels using continuous-flow breakthrough curves. Zheng et al. [66] synthesized Hydrogel-I using sodium alginate and modified alkaline lignin (MAL) as raw materials. FTIR characterization was employed to confirm the successful modification, revealing that Hydrogel-I is rich in strong complexing groups such as NH/-NH_2_, C=S, and C-S. Their research found that under the conditions of a lower influent flow rate (0.159 L/min), a low initial concentration (5 mg/L), and a higher bed depth (60 cm), the mass-transfer zone was fully expanded. Among them, the adsorption capacity and removal efficiency of the downflow mode were 2.33 times and 0.07 times higher than those of the upflow mode, respectively (with a rate constant kAB = 0.0034 L/(mg/min) and an adsorption capacity N_0_ = 678 mg/L). Similarly, Wang et al. [67] prepared Hydrogel-II by modifying lignin with Trimercapto-s-triazine trisodium salt (TMT). SEM and FTIR analyses verified the successful functionalization of lignin and the morphological stability of the hydrogel, ensuring its structural integrity during dynamic column adsorption. Under the bottom-feeding conditions with an influent flow rate of 0.159 L/min, an initial concentration of 10 mg/L, and a filling height of 40 cm, Hydrogel-II achieved an optimal bed adsorption capacity (q_0_) of 42.2 mg/g. Both studies confirmed the potential for large-scale regeneration of the materials through three consecutive adsorption–desorption cycles (Figure 4a).

To overcome the performance bottleneck of pure polymer networks, the introduction of inorganic nanomaterials represents a cutting-edge direction in composite design. Ren et al. [68] incorporated organic montmorillonite (OMMT) into sodium lignosulfonate-based hydrogels (LS-g-P(AA-co-AM)/OMMT) via free radical polymerization. A combination of SEM-EDS, XPS, XRD, and FTIR systematically confirmed the successful intercalation of the polymer into OMMT layers and the uniform distribution of active sites. These structural features provide a solid foundation for the dual chemical adsorption mechanisms of ion exchange and coordination complexation in this organic–inorganic hybrid network, which greatly increased the maximum monolayer adsorption capacities for Pb(II), Ni(II), and Mn(II) to 575.5, 326.67, and 282.82 mg/g, respectively. More broadly, the application scenarios of nanocomposite hydrogels have been extended from water treatment to complex soil environments. Shaghaleh et al. [69] developed a three-dimensional network (NCLMH) integrating MIL-100(Fe) nanomaterials and TEMPO-cellulose/lignin. Through comprehensive characterizations including zeta potential, SEM-EDS, XPS, XRD, and FTIR, the successful integration of the MOF nanoparticles into the biopolymer matrix and the resulting surface charge optimization were verified. Driven by these structural advantages, at pH 6.5, the adsorption capacities of this material for Pb(II) and Cu(II) are 416.39 mg/g and 133.98 mg/g, respectively. In the remediation experiment on greenhouse farmland, with only 0.5% addition of NCLMH, the bioavailability of lead and copper decreased by 85.9% and 74.5%, respectively, within 45 days. Combined with multi-pulse soil-water flushing technology, the NCLMH–sand column system removes lead and copper at rates of 96.5% and 85.4%, respectively, significantly inhibiting the phytotoxicity and bioaccumulation of heavy metal ions in wheat (Figure 4b).

In addition to the external component composite, exploring the molecular structural heterogeneity of lignin itself is also key to improving adsorption performance. Liu et al. [70] confirmed the value of fractional extraction and directional lignin separation. Guided by SEM, FTIR, and BET characterizations to map the morphological and chemical heterogeneity, they fractionated crude bagasse lignin into three fractions, L1, L2, and L3, and found that the L2 fraction retained the highest molecular weight and the highest proportion of high-frequency aliphatic hydroxyl groups due to strong self-polycondensation. Moreover, L2 and L3 both exhibited porous structures, with specific surface areas of 2.83 m^2^/g and 10.75 m^2^/g, respectively. Such a specific functional group distribution endows L2 and L3 with significantly higher Pb(II) adsorption capacities (6.46 mg/g and 7.56 mg/g, respectively) than crude lignin, revealing the decisive roles of molecular weight and hydroxyl density in chemical complexation.

Ultimately, material-level innovations are accelerating toward in-depth engineering governance. Khanam et al. [71] developed a dual-stage packed tower system, more closely aligned with industrial practical applications, consisting of a polyacrylamide-grafted lignin hydrogel (AM-g-AL) and a Fuller’s earth carboxymethyl cellulose-activated carbon composite (FE-CMC-AC) in series. This system not only relies on the complexation of surface carboxyl groups but also ingeniously leverages the precipitation mechanism by which Pb(II) forms insoluble Pb(OH)_2_ and PbCO_3_ at neutral pH (6–8). During dynamic operation, the dynamic adsorption capacities of AM-g-AL and FE-CMC-AC were 111.2 mg/g and 278.45 mg/g, respectively, with a cumulative capacity of 715.13 mg/column in the dual-column system. This fixed-bed design, characterized by multi-material synergy and coexisting mechanisms, provides a highly scalable systematic solution for addressing complex operating conditions ranging from low-concentration groundwater to high-concentration industrial wastewater (Figure 4c).

In summary, the research on the adsorption of Pb(II) using lignin-based hydrogels has systematically constructed an efficient, stable, and practically applicable heavy metal removal system through precise functional group design, multi-scale composites (polymers, natural polymers, inorganic nanomaterials), and engineering exploration from static to dynamic states. This has successfully expanded the application scope from water treatment to soil remediation.

#### 5.2.2. Cu

Research on lignin-based hydrogels for copper ion (Cu(II)) treatment has shown a systematic evolution from single-component systems to multi-component composites, from pure water purification to soil ecological remediation, and from single-pollutant removal to synergistic control of multiple pollutants. This process has not only deepened the understanding of the heavy metal adsorption mechanism but also achieved leapfrog development in material structure design and expanded application scenarios.

The introduction of inorganic nanomaterials creates a multi-level system of adsorption-active sites within the lignin-based hydrogel network, effectively addressing the issues of insufficient strength and the single-site distribution in organic networks. Sun et al. [72] prepared a superabsorbent hydrogel by mixing lignin and montmorillonite, yielding a synergistic effect through the ion-exchange capacity of the layered silicates and the chemical complexation of lignin functional groups. Structural and compositional analyses via SEM, FTIR, XPS, and XRD confirmed the formation of an organic–inorganic hybrid network, which underpins the maximum adsorption capacity of this system as it reaches 1.17 mmol/g. The adsorption process increases rapidly within the first five hours and conforms to the pseudo-second-order kinetic model and the Freundlich isotherm model, indicating that adsorption is driven by surface heterogeneity, controlled by a multilayer mechanism, and dominated by ion exchange. This finding echoes the study by Shen et al. [73] on magnetic carboxymethylated lignocellulose beads prepared by ionic liquid dissolution. Characterizations including TEM, PXRD, SEM, and FTIR revealed that the introduction of lignin effectively maintains the porous structure and increases the composite system’s hydroxyl value (and thus its porosity). The maximum adsorption capacities of the material for Cu^2+^ and Pb^2+^ are 0.144 mmol/g and 0.161 mmol/g, respectively, further confirming that constructing a high-porosity network to improve the accessibility of active sites is key to enhancing Cu^2+^ capture efficiency. Based on the comprehensive findings of these two studies, an “effect window” exists in the inorganic–organic composite system: an appropriate amount of inorganic components provide structural support and exchange sites, while excess lignin will induce site agglomeration due to steric hindrance. This rule provides a theoretical basis for optimizing the composite ratio.

The “total component utilization” strategy shifts the research focus from extracted lignin to the unseparated biomass as a whole, thereby achieving a profound integration of resource utilization and environmental governance. Shan et al. [74] employed a one-pot method to successfully synthesize a novel hydrogel adsorbent from cellulose, lignin, and hemicellulose derived from wheat straw. Evidence from SEM, FTIR, and XPS confirmed that the breakthrough of this study lies in the use of polyacrylic acid chains to closely cross-link cellulose microfibers with lignin/hemicellulose. This approach not only maintains the native structure of biomass but also endows the material with excellent water retention capacity and heavy-metal capture capacity. The adsorption behavior of this adsorbent follows the Freundlich model; is driven by spontaneous, endothermic processes; and is mainly dominated by ion exchange. This strategy not only reduces carbon emissions by fully utilizing resources but also enhances the economic value of straw by simplifying component separation steps. This concept was further deepened in the study by Wang et al. [75]. By modifying lignosulfonates in corn straw via hydrothermal treatment, the adsorption kinetics in complex wastewater were significantly improved. Analyses using SEM-EDS, TGA, FTIR, and XPS verified the successful modification and the resulting structural evolution, which enabled the modified hydrogel to achieve adsorption capacities of 450.3 mg/g for Cu^2+^ and 475.4 mg/g for Pb^2+^. In actual wastewater treatment, this adsorbent efficiently removed multiple metals within 2 h (e.g., 83% lead, 72% cadmium, 87% copper, 95% chromium), demonstrating the feasibility of the total component utilization strategy in large-scale industrial environmental governance.

As material properties mature, the application scenarios of lignin-based hydrogels have expanded from static water treatment to the complex field of soil remediation. Ding et al. [76] synthesized a sodium lignosulfonate/carboxymethyl sea-buckthorn seed gum composite hydrogel (SL-Cg-g-PAA) using lignin and sea-buckthorn seed gum as precursors. A suite of characterization techniques (SEM-EDS, TGA, FTIR, XPS, and zeta potential) confirmed the successful synthesis and elucidated the surface charge and structural properties of the composite. This material not only achieves a static adsorption capacity of 172.41 mg/g but also demonstrates stable immobilization capability in complex soil matrices. Through electrostatic attraction, complexation, and cation exchange, SL/Cg-g-PAA significantly reduced the bioavailability of heavy metal ions, decreased the DTPA-extractable copper content by 14.1%, blocked the migration of heavy metal ions into crops, and reduced the copper contents in leaves and roots of pak choi by 55.19% and 36.49%, respectively. This achievement marks a shift in the research on lignin-based hydrogels from “pollutant removal” to “ecological risk control”.

The current research frontier focuses on synergistic removal and selective regulation in coexistence systems involving multiple pollutants. When addressing complex pollution, researchers have proposed two distinct strategies: the first is to promote mutual enhancement through chemical coupling between pollutants. For example, Dai et al. [77] designed a novel polysaccharide-based composite hydrogel (HBPEC/SA/lignin hydrogel) for phosphorus removal from wastewater and for enhanced adsorption of copper ions (Cu^2+^). ATR-IR and SEM analyses provided evidence for the hydrogel’s structure and the role of adsorbed phosphorus as bridging sites, which significantly enhanced the capture of Cu^2+^. This system not only exhibits excellent resistance to interference from coexisting anions (except HCO_3_^−^), but also skillfully utilizes adsorbed phosphorus as bridging sites, significantly enhancing the capture of Cu^2+^. Phosphorus-containing adsorbents increase the adsorption capacity for Cu^2+^ from 10.49 mg/g to 14.51 mg/g. In addition, its thermal-responsive property enables rapid, efficient desorption of the material with a small amount of eluent, greatly improving its engineering applicability. The second strategy is to construct a multifunctional independent site system to avoid competition. For instance, Li et al. [78] synthesized a hybrid hydrogel adsorbent (HPAS) using lignosulfonamide, hyperbranched polyamidoamine, and polyethylene glycol diglycidyl ether. FTIR, XPS, and SEM characterizations revealed the hierarchical porous and hyperbranched network structure of HPAS, which enables spatial and mechanistic decoupling for different pollutants: it exhibits an ultrahigh adsorption capacity of up to 5120 mg/g for Congo red (CR), which mainly relies on hydrogen bonding, electrostatic attraction and π–π stacking; meanwhile, the maximum adsorption capacity of HPAS for Cu(II) reaches 249 mg/g, which is dominated by complexation/chelation and surface precipitation, and the performance remains stable after multiple cycles. The work of Dai et al. [77] and Li et al. [78] jointly established the theoretical framework for the synergistic treatment of multiple pollutants, effectively breaking the performance bottleneck of traditional adsorbents in complex water bodies through “chemical bridging” or “site orthogonality”.

For multi-metal competitive systems, selective regulation presents another core challenge. Liu et al. [79] successfully prepared a PAA-g-APL composite hydrogel by grafting acid-pretreated alkaline lignin onto polyacrylic acid (PAA). XPS, SEM, and EDX analyses were instrumental in elucidating the material’s composition and the spontaneous formation of a highly developed porous structure induced by phase separation between lignin and polyacrylic acid. In wastewater containing multiple heavy metal ions, this material overcomes the limitation of non-selective adsorption by conventional adsorbents and exhibits significant Pb^2+^ selectivity, with a selectivity coefficient reaching 20.22. The adsorption capacity follows the order Pb^2+^ (1.076 mmol/g) > Cu^2+^ (0.3233 mmol/g) > Cd^2+^ (0.059 mmol/g), and the molar distribution coefficient of Pb^2+^ is as high as 68%, which is significantly higher than that of Cu^2+^ (28.6%) and Cd^2+^ (3.4%). Combined with the analysis of kinetic and isotherm models, this extremely high selectivity originates from the spontaneous strong chemical affinity between the target ions and the specific sites. This achievement provides an important material design paradigm for the precise separation and extraction of specific highly toxic/high-value metals in complex electroplating wastewater or mining tailings.

#### 5.2.3. Cr

Research on lignin-based hydrogels for chromium pollution control has demonstrated a multi-dimensional technological evolution from single adsorption to integrated adsorption and detection, from water purification to soil remediation, and from physical adsorption to chemical reduction. In view of the special properties of Cr(VI), including high toxicity, strong oxidizability, and the presence of anionic forms (CrO_4_^2−^/Cr_2_O_7_^2−^), a technical path centered on “electron transfer-mediated reduction” and “directional regulation of surface charge” has emerged in this field.

**Figure 5:**
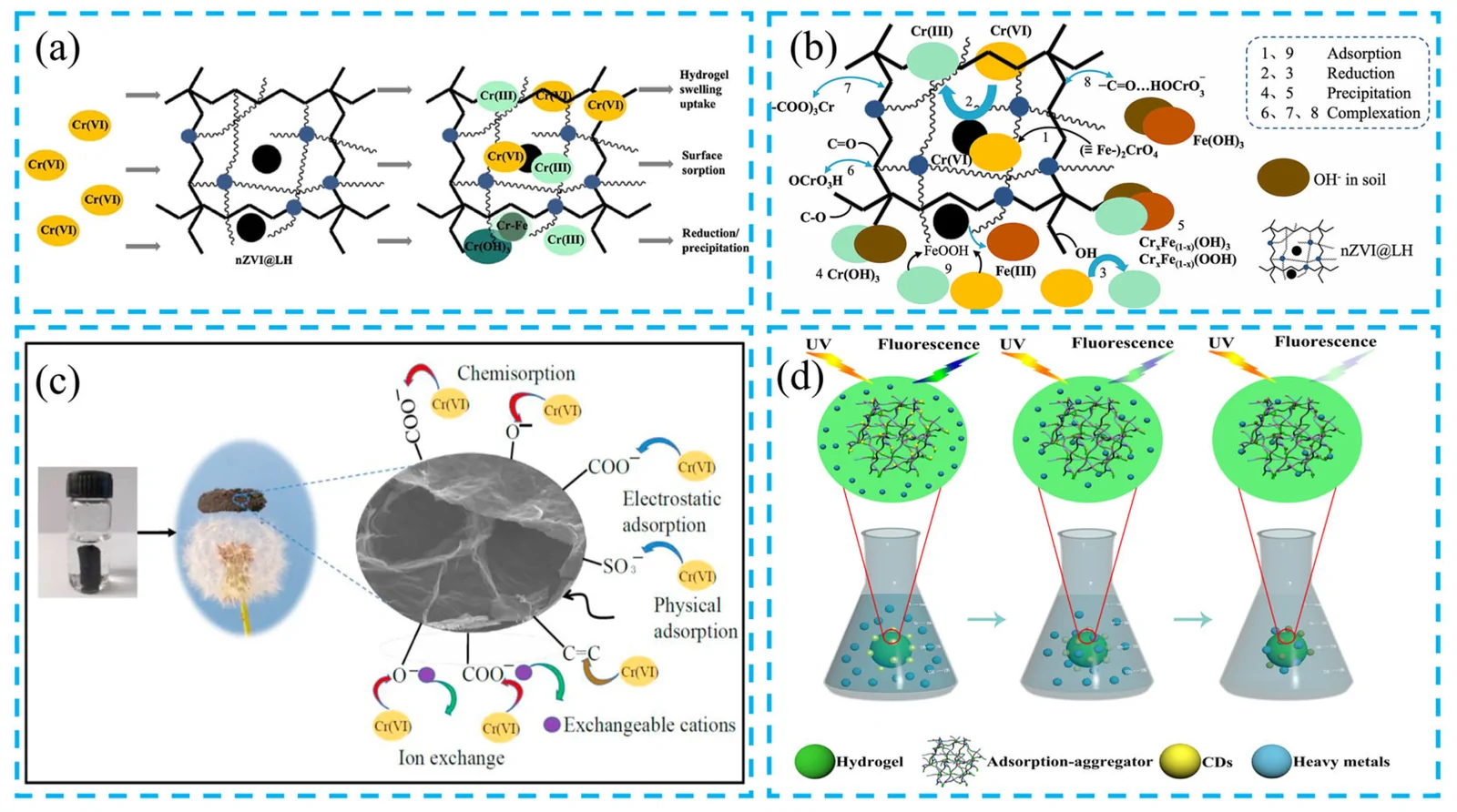
(**a**) Mechanism of efficient Cr (VI) removal by novel lignin hydrogel-supported nZVI; reproduced with permission from reference [80], Copyright 2022, Elsevier. (**b**) Remediation mechanism of nZVI@LH in Cr-contaminated soil; reproduced with permission from reference [81], Copyright 2022, Elsevier. (**c**) Possible adsorption mechanism of Cr (VI) capture by LGH; reproduced with permission from reference [82], Copyright 2021, Elsevier. (**d**) Mechanism of efficient adsorption and detection of Cr (VI) by novel fluorescent lignin-based hydrogel; reproduced with permission from reference [83], Copyright 2021, Elsevier.

The introduction of nanomaterials has successfully upgraded lignin-based hydrogels from a mere physical barrier to an active reduction–adsorption synergistic system. To overcome the drawback that nanoscale zero-valent iron (nZVI) is extremely prone to aggregation and passivation, Liu et al. developed a hydrogel-supported nZVI composite material (nZVI@LH). They verified the generality of this system in advanced water purification [80] and in situ remediation of complex industrial soil [81]. In water treatment scenarios [80], the lignin network effectively blocks nZVI aggregation through spatial confinement, thereby enhancing reaction activity. Comprehensive characterizations using SEM-EDS, FTIR, XPS, and XRD confirmed the uniform distribution of nZVI and the successful formulation of the composite network. This structural advantage translates to remarkable performance, as the removal capacity of this material for Cr(VI) reaches 310.86 mg/g Fe^0^ at pH 5.3, which is 11.6 times that of pure nZVI, exhibiting a higher acid-driven reduction efficiency under strongly acidic environments (Figure 5a). More importantly, when this system is applied to the remediation of industrial-contaminated soil [81] with a low addition amount of only 3% (*w*/*w*), the immobilization efficiency of hexavalent chromium increased from 26% with bare nZVI to more than 87% within 14 days. Solid-phase evidence from SEM-EDS, FTIR, and XRD elucidates that such performance improvement is attributed to the protection of reduction sites by the lignin hydrogel and its dominant phase transition process: nZVI@LH promotes the conversion of highly toxic water-soluble chromium (WS) to extremely stable Fe-Mn oxide-bound (OX) and organic matter-bound (OM) fractions, achieving a logical leap from “pollutant removal” to “habitat stabilization” (Figure 5b).

To achieve ultrahigh adsorption capacity, researchers constructed a system with ultrahigh-density active sites by combining high-specific-surface-area materials, such as graphene, with lignin. Sun et al. [82] addressed the dispersion challenge of graphene during the gelation process by leveraging the strong hydrophilicity of lignin sulfonate. Structural and surface analyses through FTIR, XPS, BET, TGA, and SEM-EDX verified the uniform dispersion of graphene and the formation of an interconnected, porous lignin-based graphene hydrogel (LGH). The adsorption capacity of this material for Cr (VI) reaches an impressive 1743.9 mg/g. This ultrahigh adsorption capacity originates from the synergistic chelation effect between the ultrahigh specific surface area of graphene and the abundant oxygen-containing functional groups of lignin. Furthermore, the material exhibits outstanding cycling stability, with recovery efficiency exceeding 88% after five cycles, demonstrating the potential for biomass-modified, carbon-based materials in extreme pollution load conditions. In terms of functional integration, the introduction of cellulose nanofibers and carbon dots has promoted the development of hydrogels toward intelligent monitoring (Figure 5c). Yuan et al. [83] prepared a lignin-based hydrogel with fluorescent properties via copolymerization. Characterizations via SEM, FTIR, XPS, and XRD not only confirmed the structural integrity of the hydrogel metric but also verified the successful introduction of the carbon dots. Driven by these engineered features, this material not only possesses an adsorption capacity of 599.9 mg/g for Cr (VI), but also achieves sensitive monitoring of Cr (VI) (with a detection limit of 11.2 mg/L) through the stable fluorescence signal of carbon dots. This integrated “adsorption-detection” design provides a novel solution for synchronizing environmental monitoring and remediation (Figure 5d).

The cationization modification strategy significantly enhances the capture capacity of materials for anionic Cr(VI) by introducing positively charged groups. Wei et al. [84] grafted acrylamide (AM) and acryloyloxyethyltrimethyl ammonium chloride (DAC) onto sodium lignosulfonate (LS) via free radical copolymerization, and the maximum adsorption capacity reached 58.86 mg/g. Spectroscopic and morphological analyses using FTIR, SEM, and XRD provided crucial mechanistic insights, revealing that the adsorption mechanism involves the coupling of electrostatic attraction, hydrogen bonding, and chemical reduction. The adsorption process follows the Langmuir isotherm, indicating monolayer adsorption. Adsorption kinetics show that the pseudo-second-order kinetic model dominates the adsorption process, and the adsorption activation energy is 5.489 kJ·mol^−1^. Similarly, Boulett et al. [85] synthesized a novel lignin hydrogel from poly([2-(acryloyloxy)ethyl] trimethylammonium chloride) and sodium lignosulfonate (LS) via free radical polymerization. Supported by structural and thermal evidence from FTIR, TGA, and SEM/EDS, their study demonstrated that by adjusting the blending proportion of sodium lignosulfonate, the thermal stability and hydration capacity (up to 2000%) of the adsorbent material were significantly improved, and a Cr(VI) removal efficiency of 88.4% was achieved at an LS blending content of 5.00 wt%. Such studies indicate that by precisely regulating the charge properties of the lignin surface, the competitive interference from coexisting anions can be effectively overcome, thereby improving the material’s practicality in complex industrial wastewater.

Cyclic regeneration performance is a core indicator for evaluating the application value of engineering materials, and the regeneration efficiency of an adsorbent often depends on the strength of its internal chemical bonding. Kwak et al. [86] prepared a mixed-bead adsorbent using silk sericin (SS) and kraft lignin (KL), which exhibited excellent performance at the optimal mass ratio of 50:50. SEM-EDS and FTIR analyses verified the stable cross-linking and robust surface functionalization of the hybrid beads, which underpin their cyclic durability. Consequently, this material showed outstanding regeneration robustness in a 1 M NaOH desorption system, and still maintained 82% of its original adsorption efficiency after seven cycles. This mixed-bead adsorbent based on agricultural by-products not only reduces remediation costs but also enhances thermodynamic stability through the multi-site synergistic effect of heterogeneous surfaces, providing a sustainable technical paradigm for industrial-scale Cr (VI) remediation.

#### 5.2.4. Cd

Research on lignin-based hydrogels for cadmium pollution control has shown a systematic technological evolution from aqueous-phase adsorption to soil remediation, from single-function systems to multiple synergistic effects, and from laboratory verification to field application. As a highly toxic heavy metal pollutant with strong mobility and bioaccumulation, the governance demand for cadmium has driven continuous innovation in material design and the functional integration of lignin-based hydrogels, forming a technical system characterized by dominant chemisorption and synergistic interactions among multiple mechanisms (Table 2).

The adsorption performance of lignin-based hydrogels for Cd(II) depends largely on the network structure and functional group composition of the material. By constructing a semi-interpenetrating polymer network (semi-IPN), Ji et al. [87] cross-linked cattail leaf lignin with acrylamide. Supported by SEM, XRD, FTIR, XPS, and TG, they showed that the abundant –OH and –NH groups generate strong electrostatic attraction and chelating interactions, leading to an adsorption capacity of 287.38 mg/g for Cd(II), while maintaining structural stability and regeneration capability. To overcome the limitations of purely polymer-based networks, especially insufficient mechanical strength and constrained adsorption capacity, Sheng et al. [88] adopted an organic–inorganic hybridization strategy to combine lignin with montmorillonite (MMT). SEM-EDS, XRD, and FTIR results indicated that introducing MMT not only increased mechanical strength and specific surface area, but also increased the maximum adsorption capacities of the material for Cd(II) and Zn(II) to 587.14 mg/g and 297.13 mg/g, respectively. This monolayer adsorption mode, dominated by chemical bonding, endows the material with excellent desorption resistance and reusability. Apart from the pursuit of absolute adsorption capacity, practical wastewater treatment emphasizes adsorption kinetics in multi-component systems. For this purpose, Ma et al. [89] synthesized a double-network hydrogel with a macroporous structure (RH-CTS/PAM) using rice husk lignin. XPS evidence and morphological design supported the formation of a bioinspired macroporous pathway that significantly shortens the mass-transfer distance. In a single-component system with an initial concentration of 200 mg/L, adsorption equilibrium can be reached in only 10 min, and the theoretical maximum capacities for Pb (II), Cu (II), and Cd (II) are 374.32 mg/g, 196.68 mg/g, and 268.98 mg/g, respectively. More importantly, in some ternary complex systems, their adsorption rates and capacities are even higher than those in binary systems, demonstrating superior selectivity and kinetic advantages in competitive adsorption environments.

The introduction of nanomaterials has provided a critical pathway for lignin-based hydrogels to overcome the bottleneck in adsorption capacity. The lignin-based hydrogel composite coated with magnetic hydroxyapatite (Fe_3_O_4_/HAP@lh) synthesized by Lian et al. [90] exhibited a maximum adsorption capacity of 527.74 mg/g for Cd^2+^ in aqueous solution at an optimal dosage of 2 g/L. Characterizations including SEM-EDS, XRD, FTIR, and XPS confirmed the successful composite construction and the functional interactions between Cd^2+^ and the active sites. In addition, by leveraging magnetic separation, the adsorption efficiency remained above 63% after four cycles, and the recovery rate exceeded 98%. However, when moving from homogeneous aqueous phases to complex soil environments, the conventional “adsorption–separation” mode is no longer applicable. Achieving in situ, long-term immobilization of cadmium becomes the central challenge. To address this issue, Liu et al. [91] developed a highly stable ferrous sulfide nanoparticle@lignin hydrogel (FeS@LH). SEM-EDS, XRD, and XPS confirmed the composite structure and the formation of Cd-binding phases. Unlike passive adsorption in aqueous phases, this system demonstrated a unique “active desorption–secondary capture” chemical intervention mechanism in soil: the hydrogel actively promotes the desorption of immobilized Cd^2+^ by releasing H^+^, which is then re-captured with an adsorption capacity of up to 833.3 g·kg^−1^. The core solidification mechanism fundamentally transcends single physical adsorption, with cadmium sulfide (CdS) formed by chemical precipitation accounting for an absolute dominance (84.06%), supplemented by lignin complexation (13.19%), direct nanoparticle adsorption (2.15%), and swelling effects (0.61%). In a 7-day treatment experiment, this material significantly reduced the total cadmium, surfactant-soluble cadmium, and immobilized cadmium contents in moderately and severely polluted paddy soils (with reduction rates of 22.4–49.6%, 13.5–68.6%, and 16.6–40.1%, respectively). Simultaneously, iron hydroxides formed by the oxidation of Fe^2+^ released from the system also construct a secondary adsorption barrier.

With the clarification of the cadmium immobilization mechanism, the research focus has officially shifted to field ecological restoration of farmlands and long-term safety assessment. Wei et al. [92] conducted an ecological assessment in a real-world scenario of cadmium-contaminated paddy fields. The results showed that after applying 30 g/m^2^ of FeS@LH for 30 days, the effective action distance reached 30 cm, removing 37.6% of cadmium from the soil and reducing the cadmium content in Ipomoea aquatica by 34.5%. This study was the first to systematically verify its macroecological compatibility: it not only increased total nitrogen (16.1%) and organic matter (13.8%), but also achieved a zebrafish survival rate of 98.9%, and restored the metabolic activity of the soil microbial community after treatment. After field feasibility was verified, overcoming the “easy passivation” problem of nano-restorers in complex microenvironments has become the focus of research. Ma et al. [93] further synthesized a lignin-coated nano-iron sulfide composite material (BLS-nZVI@LH). Its core innovation lies in the effective inhibition of the agglomeration and oxidation of nano-zero-valent iron by leveraging lignin’s steric hindrance. SEM-EDS, XRD, FTIR, and XPS supported the successful coating and the retained reactive features. In a continuous broccoli planting experiment, persistent FeS active sites provided ongoing redox and surface-complexation effects, resulting in Cd accumulation in crops below safety thresholds and a reduction in dietary health risk. Meanwhile, improved soil microecology led to enhanced soil enzyme activity and increased microbial community abundance.

In recent years, research on lignin-based hydrogels for cadmium (Cd) remediation has advanced beyond mere “contaminant removal” to multifunctional ameliorative materials capable of addressing complex agricultural stresses. Ma et al. [94] constructed a composite hydrogel of sodium lignosulfonate and γ-polyglutamic acid (L-GH) and combined it with foliar-applied selenium (Se), thereby proposing a novel strategy to combat the compound stress of “drought + heavy metal ions (Cd, Cu, Zn)”. SEM, XRD, FTIR, and XPS confirmed the composite structure and functional integration. Leveraging the porous lamellar structure and hydrophilic groups of the hydrogel, this system significantly enhanced soil water content, improved aggregate stability and organic carbon content, and synergistically interacted with selenium to reduce Cd and Cu concentrations in broccoli by 31.06% and 72.14%, respectively. This strategy notably increased plant photosynthetic activity, decreased malondialdehyde (MDA) content, and promoted biomass accumulation, signifying a paradigm shift in lignin hydrogels from a singular “environmental remediation agent” to an “agricultural habitat sustainable regulation material.”

**Table 2:** Lignin-based hydrogel/composite adsorbents for heavy metal remediation.

Material	Target	(Magnetic/Catalytic/Loaded)	Key Characterization	Models	Max Capacity (qmax)	Main Interaction	Ref.
CSL: chitosan/lignin hydrogel	Pb(II), Cu(II)	/	FTIR, SEM, XPS, TGA	Langmuir	Pb 139.86, Cu 98.71 mg g^−1^	Hydroxyl, amino, ether-bond network; endothermic complexation	[63]
AHL-g-PAA: acid-hydrolyzed lignin + PAA	Pb(II)	pH-responsive	FTIR, SEM, BET, XPS	Langmuir/PSO*	235 mg g^−1^	Increased density of complexation sites	[64]
UCHy: cellulose–lignin composite	Pb(II)	Ultrasonic-assisted	FTIR, SEM, BET, XPS	Langmuir, Freundlich/PSO, Schott	786.16 mg g^−1^	Cavitation induces pores; non-Fickian diffusion swelling	[65]
Hydrogel-I: MAL + sodium alginate	Pb(II)/dynamic	Dynamic fixed-bed	FTIR, SEM, XPS	Thomas model (kAB, N_0_)	Bed capacity N_0_ = 678 mg L^−1^	C=S, C–S, and –NH/–NH_2_ complexation in flow	[66]
Hydrogel-II: TMT-lignin + sodium alginate	Pb(II)/dynamic	Dynamic fixed-bed	SEM, BET, XPS	/	Bed capacity q_0_ = 42.2 mg g^−1^	Morphological stability during dynamic bed operation	[67]
LS-g-P(AA-co-AM)/OMMT	Pb, Ni, Mn	Nanoclay loaded	FTIR, SEM, XRD, XPS	Langmuir (monolayer)	Pb 575.5, Ni 326.67, Mn 282.82 mg g^−1^	Intercalated layer structure; ion exchange + coordination	[68]
NCLMH: TEMPO-cellulose/lignin + MOF	Pb, Cu/soil	MOF loaded (MIL-100(Fe))	FTIR, SEM, XRD, XPS	/	Pb 416.39 mg g^−1^	Surface charge optimization; MOF active site integration	[69]
Fractionated bagasse lignin (L2, L3)	Pb(II)	Directional separation	GPC, ^29^Si NMR, ^31^P NMR, XPS	/	L3 7.56 mg g^−1^	High MW and high-frequency aliphatic –OH dominate	[70]
Lignin + montmorillonite superabsorbent	Cu(II)	Nanoclay loaded	FTIR, SEM, XRD, XPS	Freundlich/PSO	1.17 mmol g^−1^	Organic–inorganic window effect; ion exchange dominated	[72]
Magnetic carboxymethylated lignocellulose	Cu, Pb	Magnetic	FTIR, SEM, XRD, VSM	/	Cu 0.144, Pb 0.161 mmol g^−1^	Porous structure maintenance; increased hydroxyl value	[73]
Total component wheat straw hydrogel	Cu(II)	Total biomass utilization	FTIR, SEM, XRD, XPS	Freundlich	Spontaneous, endothermic	Native biomass structure preserved; strong water retention	[74]
Hydrothermally modified corn straw	Cu, Pb/multi	Total biomass utilization	FTIR, SEM, BET, XPS	/	Cu 450.3, Pb 475.4 mg g^−1^	Total component strategy simplifies separation	[75]
SL-Cg-g-PAA: SL + sea-buckthorn gum	Cu(II)/soil	Soil amendment	FTIR, SEM, XPS	/	172.41 mg g^−1^; crop Cu uptake reduced by 55.19%	Electrostatic attraction, cation exchange, complexation	[76]
HBPEC/SA/lignin: polysaccharide-based	Cu(II), P	Thermal-responsive	FTIR, SEM, XPS	/	Cu cap increased to 14.51 mg g^−1^	“Chemical bridging” (adsorbed P serves as bridge for Cu)	[77]
HPAS: lignosulfonamide + hyperbranched	Cu(II), CR	Multifunctional sites	FTIR, SEM, XPS	/	Cu 249, CR 5120 mg g^−1^	“Site orthogonality” avoids competition	[78]
PAA-g-APL: alkaline lignin + PAA	Pb, Cu, Cd	High selectivity	FTIR, SEM, XPS	/	Pb 1.076 mmol g^−1^ (selectivity = 20.22)	Phase separation induces pores; specific chemical affinity	[79]
nZVI@LH: hydrogel-supported nZVI	Cr(VI)/water	NP loaded (nZVI)/catalytic	SEM, TEM, XRD, XPS	/	310.86 mg g^−1^ Fe^0^ (11.6× bare)	Spatial confinement blocks nZVI aggregation; enhanced reduction	[80]
nZVI@LH: hydrogel-supported nZVI	Cr(VI)/soil	NP loaded (nZVI)/catalytic	SEM, XRD, XPS, BCR sequential extraction	/	Immobilization > 87% in 14 days	Converts water-soluble Cr to stable Fe-Mn/OM bound fractions	[81]
LGH: lignin-based graphene hydrogel	Cr(VI)	Graphene loaded	FTIR, Raman, SEM, XPS	/	1743.9 mg g^−1^	Synergistic chelation (high surface area + lignin O-groups)	[82]
Fluorescent composite (lignin + C-dots)	Cr(VI)	Fluorescent C-dots loaded	FTIR, TEM, XPS, fluorescence spectroscopy	/	599.9 mg g^−1^; LOD 11.2 mg L^−1^	Synchronized “adsorption-detection”	[83]
Cationic LS + AM + DAC copolymer	Cr(VI)	Cationic modification	FTIR, SEM, XPS, zeta potential	Langmuir/PSO	58.86 mg g^−1^	Cationization overcomes anion repulsion; electrostatic + reduction	[84]
Cationic poly(…) + LS	Cr(VI)	Cationic modification	FTIR, SEM, XPS, swelling ratio	/	Removal 88.4%	Hydration up to 2000%; directional charge regulation	[85]
SS/KL: silk sericin + kraft lignin	Cr(VI)	Agricultural by-products	FTIR, SEM, XPS, zeta potential	/	Maintained 82% after 7 cycles	Multi-site synergy of heterogeneous surfaces	[86]
Semi-IPN cattail leaf lignin + acrylamide	Cd(II)	Semi-IPN	FTIR, SEM, XPS, TGA	/	287.38 mg g^−1^	Strong electrostatic attraction and chelating interactions	[87]
Lignin + montmorillonite (MMT) hybrid	Cd, Zn	Nanoclay loaded	FTIR, SEM, XRD, XPS	Langmuir (monolayer)	Cd 587.14, Zn 297.13 mg g^−1^	Monolayer chemisorption; increased mechanical strength	[88]
RH-CTS/PAM: rice husk double-network	Cd, Pb, Cu	Macroporous	FTIR, SEM, XPS, BET	/	Cd 268.98 mg g^−1^; eq. in 10 min	Bioinspired macroporous pathway shortens mass transfer	[89]
Fe_3_O_4_/HAP@lh: magnetic HAP + lignin	Cd(II)	Magnetic/NP loaded	FTIR, SEM, XRD, VSM	/	527.74 mg g^−1^	Magnetic separation + functional complexation	[90]
FeS@LH: ferrous sulfide NP @ lignin	Cd(II)/soil	NP loaded (FeS)/catalytic	SEM, TEM, XRD, XPS	/	833.3 g kg^−1^	“Active desorption-secondary capture”; CdS precipitation	[91]
FeS@LH: field ecological assessment	Cd(II)/soil	NP loaded (FeS)	Soil enzyme activity, microbial community analysis	/	Removes 37.6% Cd from field	Restores metabolic activity of soil microbial community	[92]
BLS-nZVI@LH: lignin-coated nZVI	Cd(II)/soil	NP loaded (nZVI)/catalytic	SEM, TEM, XRD, XPS	/	Reduces Cd dietary health risk	Lignin steric hindrance prevents nZVI agglomeration/oxidation	[93]
L-GH: SL + γ-PGA + foliar Se	Cd, Cu, Zn	Agricultural regulation	FTIR, SEM, plant physiological indices	/	Broccoli Cd reduced by 31.06%	Synergistic defense vs. drought and metal stress	[94]

Note: PAA (poly(acrylic acid)), AM (acrylamide), MAL (Mannich-aminated lignin), TMT (trimercapto-s-triazine), LS (lignosulfonate), OMMT (organically modified montmorillonite), MOF (metal–organic framework), SL (soda lignin), APL (alkaline peroxide lignin), nZVI (nanoscale zero-valent iron), HAP (hydroxyapatite), γ-PGA (poly(γ-glutamic acid)), IPN (interpenetrating polymer network), NP (nanoparticle).

## 6. Conclusions and Prospective Research Directions

As a green and sustainable biomass-derived adsorbent, lignin-based hydrogels exhibit significant potential for the removal of dyes and heavy metal ions. This capability stems from their unique structure–function design, which transforms lignin from a simple biopolymer into a sophisticated, multifunctional platform. The inherent chemistry of lignin, abundant in phenolic hydroxyls, carboxyl groups, and aromatic rings, provides natural sites for multiple interactions, including electrostatic attraction, hydrogen bonding, π–π stacking, coordination, and ion exchange. To overcome the limitations of natural lignin (such as brittleness and low solubility) and to tailor its properties for complex wastewater treatment, strategic engineering is employed. Through molecular modifications (e.g., sulfonation, graft copolymerization) and macroscopic network construction (e.g., nanofiber reinforcement, interpenetrating or magnetically responsive networks), these hydrogels develop stable, highly porous architectures. This engineered structure enhances pollutant diffusion kinetics and active site accessibility, enabling efficient and often synergistic capture of diverse contaminants. The adsorption process is typically dominated by monolayer chemisorption, as evidenced by conformity to the Langmuir isotherm and pseudo-second-order kinetics. Performance is strongly influenced by environmental factors such as pH, ionic strength, and the presence of coexisting pollutants, highlighting the importance of material design in achieving selectivity and stability under real-world conditions.

Future research should focus on the in-depth integration of material functionalization and performance optimization, and on developing multifunctional, smart-responsive lignin-based hydrogels to achieve integrated “adsorption–detection–degradation” treatment. The introduction of magnetic nanoparticles, conductive polymers, or carbon-based materials can improve the separation, recovery, and environmental adaptability of the material; the construction of molecular imprinting and specific recognition structures is expected to enhance selective adsorption toward target pollutants. Further elucidating the microscopic adsorption mechanism with advanced characterization methods (e.g., in situ XPS, EXAFS, molecular dynamics simulation, DFT) and establishing the structure–performance relationship of materials will provide theoretical guidance for the rational design of high-efficiency adsorbents.

While lignin-based hydrogels show immense promise in the laboratory, their translation to industrial wastewater treatment is contingent upon overcoming a series of interconnected practical challenges. First, the inherent variability of industrial lignin feedstocks, which differ by pulping process and plant source, undermines the batch-to-batch consistency required for large-scale synthesis. Second, the material must demonstrate sufficient mechanical integrity and long-term chemical stability to withstand the hydrodynamic stress and complex composition of real wastewaters in continuous-flow systems. Third, the economic and environmental viability of the process hinges on efficient adsorbent regeneration, sustainable management of concentrated waste streams, and a clear end-of-life strategy for spent materials. Fourth, environmental and safety concerns, particularly regarding the potential leaching of toxic cross-linkers or embedded nanoparticles, must be preemptively addressed through greener chemistries and rigorous testing. Finally, non-trivial engineering challenges in scaling up from batch to continuous operation—such as managing pressure drop and system integration—require validation through pilot-scale studies to inform robust techno-economic and lifecycle assessments. We emphasize the need for pilot-scale validation of continuous-flow processes (fixed beds and membrane reactors), systematic techno-economic analysis, and full lifecycle assessment (LCA) to address challenges including feedstock variability, long-term operational stability, and safe disposal of spent adsorbents. Validation in real industrial wastewater (e.g., electroplating and textile printing effluents) is highlighted as the ultimate criterion for practical application. These expanded perspectives cover both fundamental research and engineering translation gaps, providing a clearer direction for future studies.

Crucially, moving from idealized single-solute models to true co-contaminant systems represents a major frontier for future investigations. Since simultaneous removal is chemically more complex than single-solute adsorption, it is imperative to systematically explore competitive and cooperative adsorption behaviors, as well as the specific selectivity of hydrogels in multi-component environments. Future evaluations must account for the complex interference from coexisting ions, natural organic matter (NOM), and fluctuating ionic strengths. Meanwhile, the optimization of continuous-flow processes, such as fixed beds and membrane reactors, alongside the evaluation of cycling and regeneration stability, will facilitate the smooth transition of lignin-based hydrogels from laboratory research to industrial application. Ultimately, validating adsorption performance directly in real industrial wastewater will be the indispensable touchstone for providing sustainable solutions for treating water bodies contaminated with diverse heavy metal ions and organic dyes.

## Data Availability

All the data are provided in this manuscript.
